# Cathepsin S (CTSS) in IgA nephropathy: an exploratory study on its role as a potential diagnostic biomarker and therapeutic target

**DOI:** 10.3389/fimmu.2024.1390821

**Published:** 2024-06-24

**Authors:** Shaojie Fu, Meiyan Wu, Yanli Cheng, Yan Guan, Jinyu Yu, Xueyao Wang, Sensen Su, Hao Wu, Fuzhe Ma, Yan Zou, Shan Wu, Hongzhao Xu, Zhonggao Xu

**Affiliations:** ^1^ Department of Nephrology, The First Hospital of Jilin University, Changchun, China; ^2^ Department of Nephrology, Meihe Hospital, The First Hospital of Jilin University, Meihekou, China; ^3^ Department of Nephrology, Meihekou Central Hospital, Meihekou, China; ^4^ Center for Renal Pathology, The First Hospital of Jilin University, Changchun, China; ^5^ Department of Cardiac Ultrasound, The First Hospital of Jilin University, Changchun, China

**Keywords:** IgA nephropathy, cathepsins, causal inference, Mendelian randomization study, virtual screening

## Abstract

**Introduction:**

IgA nephropathy (IgAN), a prevalent form of glomerulonephritis globally, exhibits complex pathogenesis. Cathepsins, cysteine proteases within lysosomes, are implicated in various physiological and pathological processes, including renal conditions. Prior observational studies have suggested a potential link between cathepsins and IgAN, yet the precise causal relationship remains unclear.

**Methods:**

We conducted a comprehensive bidirectional and multivariable Mendelian randomization (MR) study using publicly available genetic data to explore the causal association between cathepsins and IgAN systematically. Additionally, immunohistochemical (IHC) staining and enzyme-linked immunosorbent assay (ELISA) were employed to evaluate cathepsin expression levels in renal tissues and serum of IgAN patients. We investigated the underlying mechanisms via gene set variation analysis (GSVA), gene set enrichment analysis (GSEA), and immune cell infiltration analysis. Molecular docking and virtual screening were also performed to identify potential drug candidates through drug repositioning.

**Results:**

Univariate MR analyses demonstrated a significant link between increased cathepsin S (CTSS) levels and a heightened risk of IgAN. This was evidenced by an odds ratio (OR) of 1.041 (95% CI=1.009–1.073, *P*=0.012) as estimated using the inverse variance weighting (IVW) method. In multivariable MR analysis, even after adjusting for other cathepsins, elevated CTSS levels continued to show a strong correlation with an increased risk of IgAN (IVW *P*=0.020, OR=1.037, 95% CI=1.006–1.069). However, reverse MR analyses did not establish a causal relationship between IgAN and various cathepsins. IHC and ELISA findings revealed significant overexpression of CTSS in both renal tissues and serum of IgAN patients compared to controls, and this high expression was unique to IgAN compared with several other primary kidney diseases such as membranous nephropathy, minimal change disease and focal segmental glomerulosclerosis. Investigations into immune cell infiltration, GSEA, and GSVA highlighted the role of CTSS expression in the immune dysregulation observed in IgAN. Molecular docking and virtual screening pinpointed Camostat mesylate, c-Kit-IN-1, and Mocetinostat as the top drug candidates for targeting CTSS.

**Conclusion:**

Elevated CTSS levels are associated with an increased risk of IgAN, and this enzyme is notably overexpressed in IgAN patients’ serum and renal tissues. CTSS could potentially act as a diagnostic biomarker, providing new avenues for diagnosing and treating IgAN.

## 1 Introduction

Immunoglobulin A nephropathy (IgAN) is one of the most prevalent glomerulonephritis worldwide. It poses a substantial risk of poor prognosis, often culminating in end-stage renal failure (ESRD) over a patient’s lifetime (1). The prevalence of IgAN exhibits variations across ethnicities, with higher rates observed in Asian populations compared to Caucasians. These discrepancies arise from disparities in primary care accessibility and the criteria for conducting renal biopsies (2). Notably, patients with IgAN exhibit considerable heterogeneity in clinical manifestations, renal progression, and long-term outcomes. Over 20–30 years, approximately 30–40% of individuals with IgAN progress to ESRD (3, 4). The intricate pathophysiology of IgAN remains largely elusive, resulting in limited effective treatments and reliable biomarkers for disease progression. Consequently, an urgent need exists to identify specific biomarkers and potential therapeutic targets for IgAN.

Proteases are enzymes that orchestrate the irreversible breakdown of proteins via peptide bond hydrolysis, contributing significantly to maintaining normal homeostasis. In mammals, proteases are classified into threonine, serine, cysteine, metallo, and aspartyl proteases based on their structural and catalytic characteristics (5). Among these, cysteine proteases, belonging to the cathepsin family, have attracted considerable attention due to their critical roles in a wide array of biological and pathophysiological processes. The cathepsin family is characterized by variations in structural features, expression profiles, distribution, localization, biochemical properties, and regulation of their activity. Importantly, these proteases are under various regulatory mechanisms to control potentially harmful proteolytic activity, especially in relation to kidney diseases.

Recent investigations have unveiled the crucial roles played by several cathepsins, such as cathepsin B, cathepsin D, cathepsin K, and cathepsin S (CTSS), in either promoting or suppressing various kidney diseases, including diabetic nephropathy, acute kidney injury, chronic kidney disease, and renal ischemia/reperfusion injury (6–9). However, studies exploring the relationship between different cathepsins and IgAN remain exceedingly scarce. Only Zhao et al. have reported higher renal tissue and serum expression of CTSS in pediatric patients with IgAN compared to normal individuals (10). Thus, it is paramount to systematically investigate the causal association between distinct cathepsins and the risk of IgAN.

**Figure 1:**
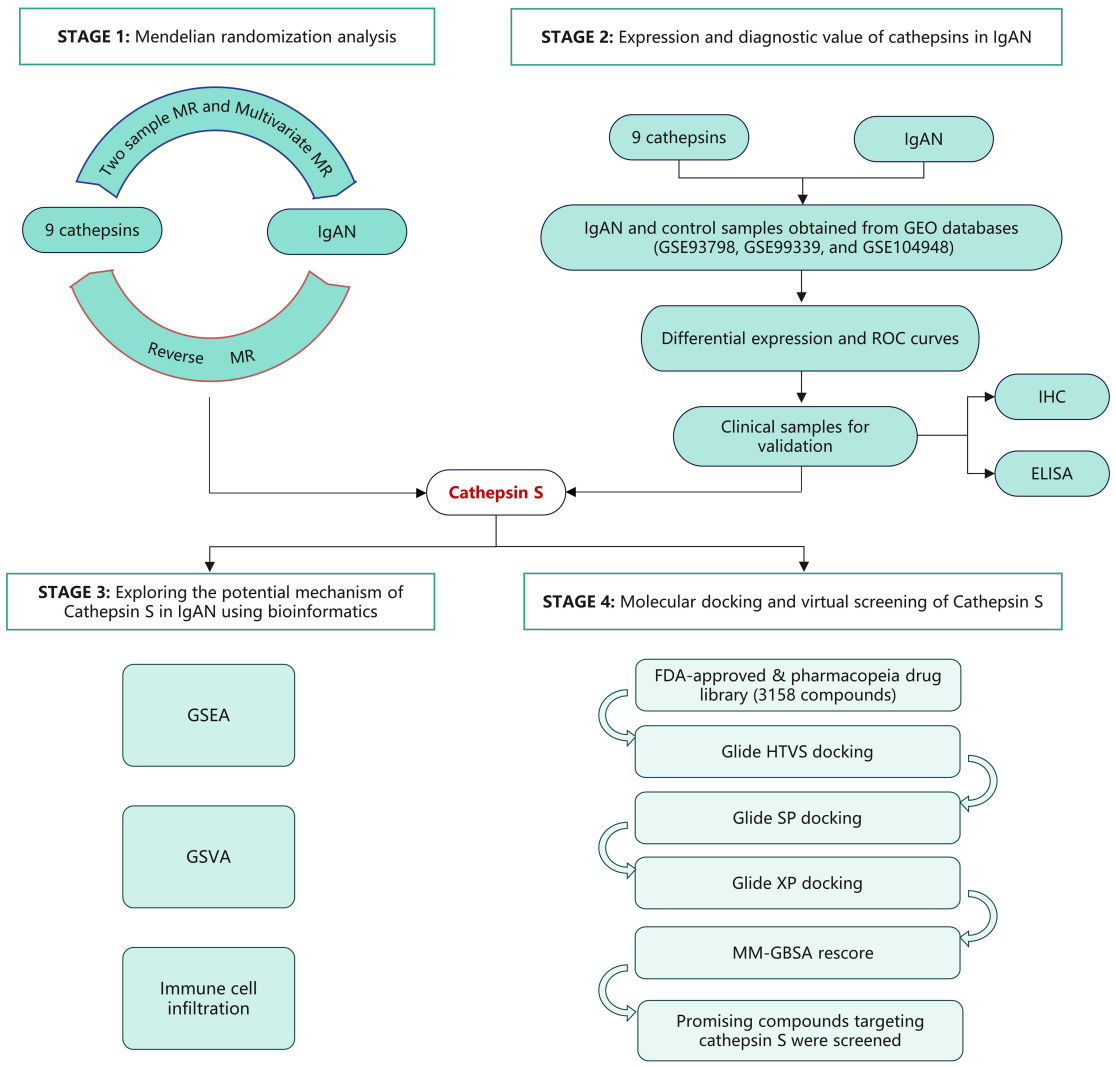
The flow chart of this study.

Mendelian randomization (MR) leverages genetic variants as instrumental variables (IVs) to infer causal relationships between exposure and outcome, utilizing data from genome-wide association studies (GWAS) (11). In this study, we employed both univariable and multivariable MR methodologies to elucidate the causal effects of various cathepsins on the risk of IgAN. Our research uncovered a significant correlation between heightened CTSS levels and increased IgAN risk. To further investigate this, we analyzed the GEO database to examine CTSS expression and its diagnostic potential in IgAN. Clinical validation was pursued through immunohistochemical (IHC) and enzyme-linked immunosorbent assay (ELISA) testing, utilizing renal tissue and serum samples from IgAN patients at our center. Bioinformatic analysis was employed to delve into possible mechanisms underlying CTSS’s role in IgAN pathogenesis. Molecular docking and virtual screening were conducted to identify potential therapeutics, focusing on drugs from the FDA-approved and pharmacopeia drug libraries that might target CTSS, emphasizing established safety profiles. A flowchart detailing the study’s progression is presented in **Figure 1**.

## 2 Materials and methods

### 2.1 Data sources for GWAS

We collected genetic instruments evaluating the levels of various cathepsins (µg/L) from the INTERVAL study, which consisted of 3,301 participants of European descent (12). The INTERVAL study was approved by the National Research Ethics Service (11/EE/0538), and all participants provided informed consent. Audiences can access summary data at https://gwas.mrcieu.ac.uk. We sourced GWAS data for IgAN from the UK Biobank and FinnGen datasets. This analysis involved a substantial cohort of 477,784 individuals of European ancestry, comprising 15,587 cases and 462,197 controls. After stringent quality control and imputation procedures (13), we analyzed approximately 25 million genetic variants.

### 2.2 Selection of IVs

In MR context, IVs are genetic variants used to establish causal relationships between exposure and outcome. The validity of IVs relies on satisfying three fundamental assumptions (11):

1. They must exhibit a direct correlation with the exposure of interest. 2. They should not correlate with potential confounding factors between the exposure and outcome. 3. They should not influence the outcome through pathways other than exposure.

To meet these three core assumptions, we employed the following criteria for IV selection (14): 1. We selected single nucleotide polymorphisms (SNPs) that exhibited a genome-wide significance level association with the exposure (*P*<5×10^-6^). 2. We excluded SNPs that displayed linkage disequilibrium within a 10,000 kb window (r^2^>0.001). 3. SNPs with an F-statistic of less than 10 were excluded. 4. We excluded SNPs significantly associated with the outcome (*P*<5×10^-8^).

Detailed information regarding the included SNPs for the exposure data can be found in **Supplementary Table 1**.

### 2.3 MR analysis

We primarily utilized the Inverse Variance Weighting (IVW) method to estimate the overall effect size. To validate the robustness of our MR results, we also employed two complementary methods: MR-Egger and Weighted Median (WM). These MR analyses were conducted using the ‘Mendelian Randomization’ package (version 0.4.3) within R software (version 4.3.1) (15).

To assess the heterogeneity among the selected IVs, we applied Cochran’s Q statistic and illustrated the heterogeneity using a random effect model (11). Additionally, we used MR-Egger to investigate potential asymmetry due to the pleiotropic effects of multiple genetic variants, aiming to exclude the influence of horizontal pleiotropy (16).

To refine our analysis further, we employed the MR-PRESSO method to identify and eliminate potential horizontal pleiotropic outliers that could significantly impact the estimation results (17).

In conjunction with standard MR analyses, we conducted reverse MR analyses, treating cathepsins as the outcome and IgAN as the exposure, to investigate the possibility of reverse causality. These reverse MR analyses employed the same GWAS datasets previously mentioned. We utilized the “MendelianRandomization” software package for multivariate MR, an advanced version of the standard univariate MR (15). This multivariate approach simultaneously considers multiple cathepsins to assess their causal impact on IgAN, enabling us to determine the direct causal effect of each exposure in a singular, comprehensive analysis. A self-assessment using the STROBE-MR checklist (**Supplementary Table 2**) confirmed that the experimental procedures met the publication requirements for MR studies.

### 2.4 Exploring cathepsin expression and its diagnostic value in IgAN using the GEO Database

We collected microarray datasets containing kidney tissue samples from both IgAN patients and controls from the GEO database (http://www.ncbi.nlm.nih.gov/geo/). Specifically, we obtained data from three datasets: GSE93798, GSE99339, and GSE104948 (18). To address any potential inter-batch differences, we merged these three datasets and applied the R SVA package for correction (19).

To identify the differential expression of each cathepsin between IgAN patients and controls, we utilized the R limma package (20). This allowed us to determine which cathepsins showed significant differences in expression between the two groups. Additionally, a small sample cohort with renal tissue microarray data from Canada was included to validate the key results (21).

To evaluate the diagnostic utility of each cathepsin, we generated receiver operating characteristic (ROC) curves using mRNA expression data from a combined dataset comprising 73 samples from IgAN patients and 59 samples from control subjects.

The area under the ROC curve (AUC) served as the metric to evaluate the diagnostic efficacy of each cathepsin. We utilized the “pROC” package in R to create ROC curves. In our analysis, we regarded a two-sided *P*-value of <0.05 as indicative of statistical significance.

### 2.5 Sample collection

Collection of kidney tissues: Kidney tissues from diagnosed IgAN, membranous nephropathy (MN), minimal change disease (MCD) and focal segmental glomerulosclerosis (FSGS) patients based on renal biopsy pathology were obtained from the First Hospital of Jilin University. Normal kidney tissues were sourced from adjacent tissue regions of kidney cancer patients.

Collection of serum samples: Serum samples were obtained from patients with IgAN, MN, MCD and FSGS pathologically confirmed by renal biopsy and healthy volunteers. In addition, serum samples from patients with IgAN pathologically confirmed by renal biopsy and healthy volunteers were also collected for validation at a medical center in the Meihe region, which is 200 kilometers away from our city.

On the first morning of hospitalization, 5 ml of venous blood was collected from the elbow. These blood samples were then placed into procoagulant tubes and centrifugated at 3500 rotations per minute at a temperature of 4°C. The serum was separated and stored at -80°C for subsequent analysis. The study has been approved by the ethical committees (the First Hospital of Jilin University, approval number 2023–508).

### 2.6 Renal immunohistochemical staining

To delve deeper into the differences in cathepsin expression between IgAN and healthy kidney tissues, we carried out IHC staining. We utilized kidney samples from IgAN, MCD, MN and FSGS patients, and healthy controls. Initially, all these tissue samples were fixed in 10% formalin, paraffin-embedded, and then sliced into sections that were 5 µm thick. Following this, a series of steps, including dewaxing, rehydration, and an antigen retrieval procedure, were performed on these kidney tissue sections. The next step involved incubating the sections with a rabbit anti-human Cathepsin S antibody (diluted at 1:250, Abcam, USA) at 4°C overnight. After thorough washing, the antibodies that had bound were subjected to a reaction with horseradish peroxidase (HRP)-labeled goat anti-rabbit IgG and were visualized through DAB (3,3′-Diaminobenzidine).

Following these steps, we conducted counterstaining using hematoxylin and then captured images of the sections under a light microscope. The IHC staining was analyzed using Image-Pro Plus 6.0 software. We employed a semiquantitative scoring method that considered both staining intensity and the percentage of positively stained cells.

### 2.7 ELISA

In the ELISA procedure, we quantified the serum CTSS levels using a double antibody Sandwich ELISA technique (22). Our approach involved using a detection reagent obtained from Shanghai Cohesion, with strict adherence to the provided instructions. Here is a summary of the procedure:

Initially, 100 μl of serum and standard solutions were added to the CTSS antibody-coated 96-well microplate and incubated at room temperature for 90 min. After the incubation, the solution was discarded, and the plate was washed three times with a Wash Buffer Working Solution. Next, 100 μl of biotin-labeled detection antibody was added to each well, and the microplate was incubated at 37°C for 60 minutes. Following this incubation, the solution was again discarded, and the plate was washed three times. Subsequently, 100 μl of Streptavidin-HRP Working Solution was added to each well, and the microplate was incubated at 37°C for 45 minutes. After the second incubation, we added Tetramethylbenzidine substrate solution, initiating an enzymatic colorimetric reaction. This reaction proceeded at 37°C for 15 minutes in a light-protected environment. Subsequently, to terminate the reaction, a stop solution was introduced. We measured the optical density of the sample at 450 nm and compared it with a pre-established standard curve to calculate the final concentration. This standard curve was generated using various concentrations of CTSS, specifically at 156.25, 312.5, 625, 1250, 2500, 5000, and 10000 pg/ml, respectively. The detection range for this assay spanned from a lower limit of 156.25 pg/ml to an upper limit of 10000 pg/ml. For accuracy, all samples underwent testing in triplicate.

### 2.8 Gene set enrichment analysis and gene set variation analysis analyses for CTSS

Our study conducted GSEA and GSVA analyses for CTSS using IgAN samples obtained from the GEO database. These samples were categorized into high and low expression groups, determined by their CTSS expression levels relative to the median value. We then utilized GSEA and GSVA to assess these groups.

For GSEA, we examined the enrichment of biological process pathways by utilizing the “c5.go.bp.v7.4.symbols” gene sets sourced from the MSigDB database (23). This allowed us to identify significant associations between CTSS expression and biological processes. Additionally, we employed the GSVA algorithm to pinpoint significantly enriched KEGG pathways between the high and low-expression groups (24).

### 2.9 Immune cell infiltration

To understand the immunological characteristics of IgAN and control groups, we employed the CIBERSORT deconvolution algorithm. Subsequently, we used the R ggplot2 package to create a violin diagram, which visually represents the variances in the infiltration of 22 distinct types of immune cells within kidney tissues for both the IgAN and control groups (25). Furthermore, we conducted Spearman’s rank correlation analysis to depict the relationships between CTSS and immune-infiltrating cells. These correlations were then visualized using the ggstatsplot and ggplot2 packages in R software. Additionally, XCELL, which is another generally accepted bioinformatics algorithm for performing immune infiltration analysis at present, was also conducted as a complement (26).

### 2.10 Molecular docking and virtual screening

For molecular docking and virtual screening, we obtained the crystal structure of human CTSS (PDB ID: 4P6G) from the Protein Data Bank (PDB) database (27), with a resolution of 1.58Å. Notably, the protein structure is a homotetramer, but we exclusively utilized the A chain for our analysis. The sitemap prediction identified five sites on the protein. After careful evaluation, we selected the binding pocket with the highest hydrophobicity and score for virtual screening.

We utilized the Protein Preparation Wizard module in the Schrödinger software suite to prepare the protein structure. This process included assigning bond orders, adding hydrogens, and other necessary operations. The Prime module was employed to reconstruct any missing side chains, as needed (28). The protonation states were determined at a pH of 7.0 using PROPKA, followed by an optimization of the hydrogen bonding network. Lastly, we performed a restrained minimization of the protein employing the OPLS_4 force field. We aimed to minimize and converge the heavy atoms to a root-mean-square deviation of 0.30 Å.

We utilized the FDA-approved & pharmacopeia drug library (Targetmol) for the virtual screening, which included 3158 compounds. These compounds were imported into the Schrödinger software in 2D sdf files, and their 3D structures were prepared. Coordinate determination was performed using the Ligprep module, which relied on the OPLS_4 force field. All possible stereoisomers and associated protonation states were determined using the Epik method at pH 7.0 ± 2.0 for the tautomer generation (29). The virtual screening process encompassed four sequential steps:

1. Glide high-throughput virtual screening (HTVS). 2. Glide standard-precision (Glide SP) docking. 3. Glide extra-precision (Glide XP) docking. 4. Molecular mechanics/generalized Born and surface area solvation (MM-GBSA) calculations (30–33).

This comprehensive workflow allowed for the systematic screening and evaluation of compounds from the drug library against the selected binding pocket of the CTSS protein.

## 3 Results

### 3.1 Defining the causal relationship between various cathepsins and IgAN

**Figure 2:**
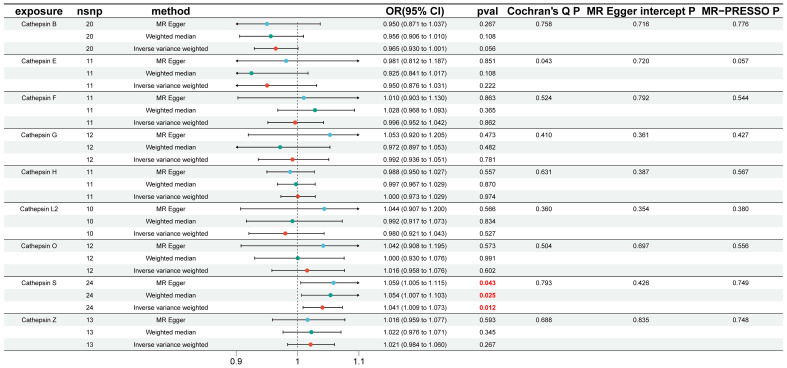
Causal association of cathepsins on IgAN estimated by univariable Mendelian randomization analysis. This figure displays the causal association of cathepsins on IgAN, as estimated by univariable Mendelian randomization analysis. Three Mendelian randomization methods, namely Inverse Variance Weighting, MR-Egger, and Weighted Median, were employed to investigate the causal relationships between nine cathepsins (cathepsin B, E, F, G, H, L2, O, S, and Z) and IgAN. Statistically significant results are highlighted in red, and the error bars indicate 95% confidence intervals.

To investigate the causal impact of different cathepsins on the risk of IgAN, we conducted two-sample MR analyses involving nine distinct cathepsins, namely cathepsin B, E, F, G, H, L2, O, S, and Z, to assess their influence on the overall risk of IgAN. Our initial univariable MR analyses, depicted in **Figure 2**, revealed a noteworthy finding. Specifically, we observed that elevated levels of CTSS were associated with an increased risk of developing IgAN, with an odds ratio (OR) of 1.041 (95% CI=1.009–1.073, *P*=0.012) when utilizing the IVW method. Complementary tests, including WM and MR-Egger methods, supported this result, with WM yielding an OR of 1.054 (95% CI=1.007–1.103, *P*=0.025) and MR-Egger reporting an OR of 1.059 (95% CI=1.005–1.115, *P*=0.043). Importantly, our analysis revealed no evidence of heterogeneity in the results, as indicated by a non-significant Cochran’s Q test (*P*=0.793). Furthermore, both the MR-Egger intercept (*P*=0.426) and the MR-PRESSO global tests (*P*=0.426) showed no signs of directional pleiotropy within these causal associations. However, it is crucial to note that we did not establish a causal relationship between other cathepsin types and IgAN, as depicted in **Figure 2**.

**Figure 3:**
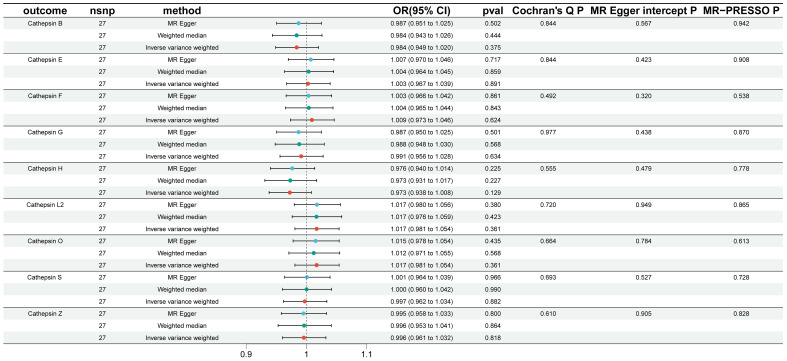
Reverse Mendelian randomization analysis performed to assess reverse causality using IgAN as the exposure and cathepsins as the outcome. In this figure, Reverse Mendelian randomization analysis was conducted to assess reverse causality, using IgAN as the exposure and cathepsins as the outcome. Similar to **Figure 2**, three Mendelian randomization methods were used to explore the causal associations between IgAN and the nine cathepsins. The error bars represent 95% confidence intervals.

To explore the possibility of reverse causality, specifically whether IgAN presence could induce changes in various cathepsin levels, reverse MR analyses were conducted. As demonstrated in **Figure 3**, our findings provided no evidence to support a causal relationship between IgAN and alterations in the levels of different cathepsins.

**Figure 4:**
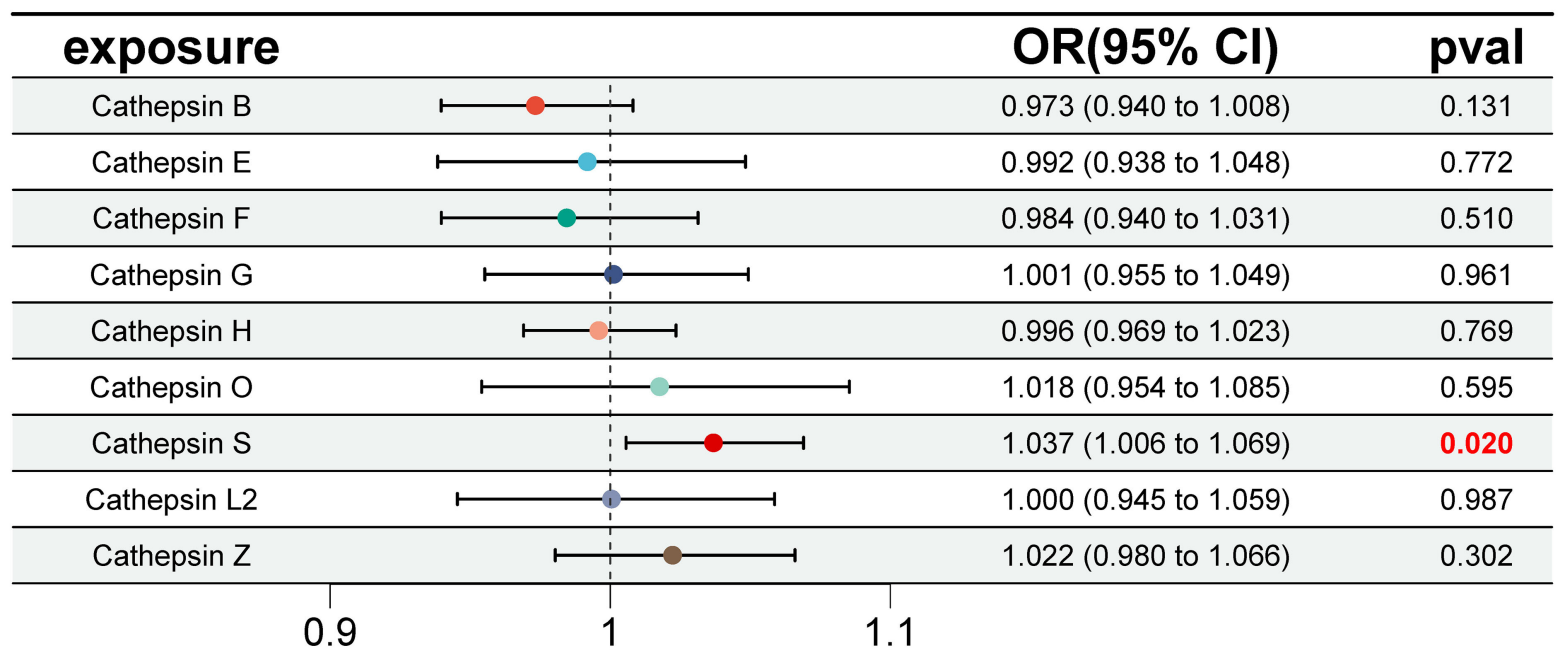
Forest plot of multivariable Mendelian randomization inverse variance weighting analysis for nine cathepsins and IgAN. This figure presents a forest plot of multivariable Mendelian randomization inverse variance weighting analysis for the nine cathepsins and IgAN. It employs the inverse variance weighting method to investigate the causal relationships between the same nine cathepsins and IgAN. Statistically significant results are highlighted in red, and the error bars indicate 95% confidence intervals.

Furthermore, we conducted multivariable MR analyses to explore the genetic predisposition involving various cathepsins concerning the risk of IgAN. As depicted in **Figure 4**, even after accounting for the presence of other cathepsin types, heightened levels of CTSS maintained a strong correlation with an elevated risk of IgAN (IVW *P*=0.020, OR=1.037, 95% CI=1.006–1.069). However, it is worth noting that we did not observe any statistically significant causal relationships between other cathepsin types and the risk of IgAN. The MR-Egger intercept analysis (*P*=0.867) provided no evidence of horizontal pleiotropy in our findings.

### 3.2 Exploring cathepsin expression and its diagnostic utility in IgAN

**Figure 5:**
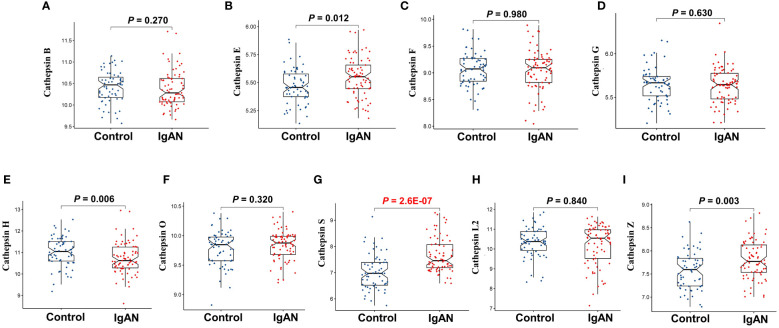
The differential expression of nine cathepsins in renal tissues of IgAN patients from GEO datasets. This figure focuses on the differential expression of cathepsin B **(A)**, cathepsin E **(B)**, cathepsin F **(C)**, cathepsin G **(D)**, cathepsin H **(E)**, cathepsin O **(F)**, cathepsin S **(G)**, cathepsin L2 **(H)** and cathepsin Z **(I)** in renal tissues of IgAN patients, using microarray data from three GEO datasets (GSE30528, GSE96804, and GSE99339). The analysis is conducted with data from 73 IgAN patients and 59 control subjects, with statistical significance set at P<0.05.

To investigate the variation in expression of various cathepsins within the renal tissues of IgAN patients, we conducted a retrospective analysis using data from 73 IgAN patients and 59 controls sourced from three GEO datasets (GSE30528, GSE96804, and GSE99339). Following the elimination of batch effects, we employed the limma package to detect differential expression patterns among nine cathepsins in both IgAN and control renal tissues. This analysis revealed statistically significant differences in the expression levels of cathepsins H, S, and Z, as illustrated in **Figure 5**, with cathepsins S showing the most significant difference (*P*=2.6E-07). Validation of CTSS expression in a small Canadian cohort (including 27 IgAN patients and 6 healthy pre-transplant living donor controls) revealed significantly higher levels of CTSS expression in IgAN patients compared to controls (*P*<0.0001), and the results are shown in **Supplementary Figure 1**.

**Figure 6:**
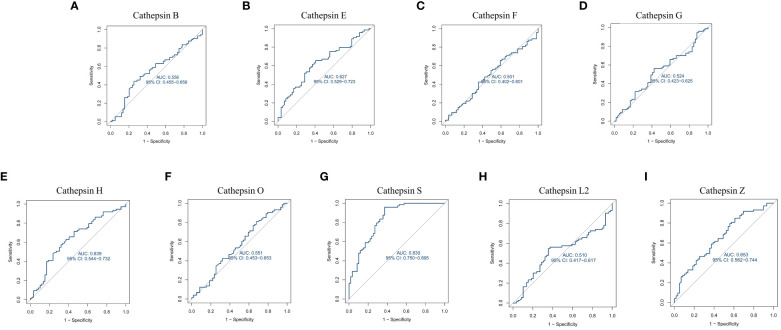
Receiver operating characteristic curve for nine cathepsins using GEO datasets. This figure is about the receiver operating characteristic (ROC) curve analysis for cathepsin B **(A)**, cathepsin E **(B)**, cathepsin F **(C)**, cathepsin G **(D)**, cathepsin H **(E)**, cathepsin O **(F)**, cathepsin S **(G)**, cathepsin L2 **(H)** and cathepsin Z **(I)** using data from GEO datasets. It evaluates the diagnostic value of each cathepsin by plotting ROC curves based on mRNA expression data from 73 IgAN patients and 59 control samples from the GEO database. The area under the ROC curve (AUC) is calculated to assess their diagnostic efficacy.

Furthermore, we constructed ROC curves for each cathepsin’s expression, as depicted in **Figure 6**. Notably, CTSS exhibited the capacity to effectively distinguish IgAN from control samples, boasting an AUC of 0.830 (95% CI 0.750–0.895). In contrast, the AUCs for the remaining cathepsins were below 0.8, indicating that CTSS holds promising diagnostic potential for IgAN.

### 3.3 Clinical validation of CTSS expression in IgAN patients

**Figure 7:**
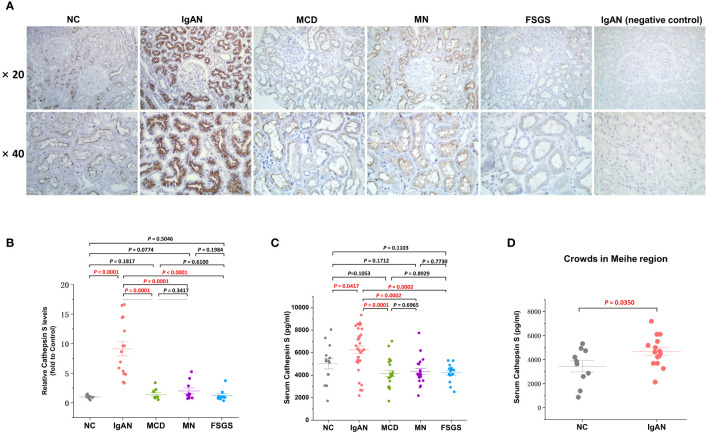
Clinical validation of the CTSS expression in kidney tissues and serum of IgAN patient. This figure provides clinical validation of CTSS expression in kidney tissues and serum of IgAN patients, MCD patients, MN patients, FSGS patients and normal controls. It includes representative images **(A)**, statistical analyses of immunohistochemical staining for CTSS **(B)**, statistical analyses of enzyme-linked immunosorbent assay for CTSS **(C)**, and statistical analyses of enzyme-linked immunosorbent assay for CTSS in an external independent cohort **(D)**.

To substantiate the differential expression of CTSS in renal tissue and serum of IgAN patients versus normal controls, and whether this differential expression is specific to IgAN compared to other primary kidney diseases such as MCD, MN and FSGS, we utilized both IHC staining and ELISA. Our IHC analysis, conducted on biopsy specimens from 15 patients with IgAN, 10 with MCD, 10 with MN, 10 with FSGS, and 9 healthy controls, revealed a predominant expression of CTSS within the microvilli located in renal tubular epithelial cells of the kidney, as depicted in **Figure 7A**. Compared to normal controls, the expression of CTSS was significantly higher in tubulointerstitial region in IgAN patients, and this high expression was unique to IgAN compared with several other primary kidney diseases as evidenced in **Figure 7B**.

Serum CTSS levels were quantified using a double antibody Sandwich ELISA, involving 34 patients with IgAN, 20 with MCD, 20 with MN, 15 with FSGS and 14 healthy controls. The results indicated that IgAN patients had notably higher levels of serum CTSS compared to healthy controls, and this high expression was also unique to IgAN compared with several other primary kidney diseases as demonstrated in **Figure 7C**. Additionally, validation of CTSS levels in the serum of a small external cohort including 15 IgAN patients and 10 healthy volunteers in the Meihe region, 200 kilometers away from our city, similarly revealed that CTSS levels in the serum of IgAN patients were significantly higher compared to controls (*P*=0.0350), as shown in **Figure 7D**.

### 3.4 Exploring mechanisms involving cathepsin S in IgAN

**Figure 8:**
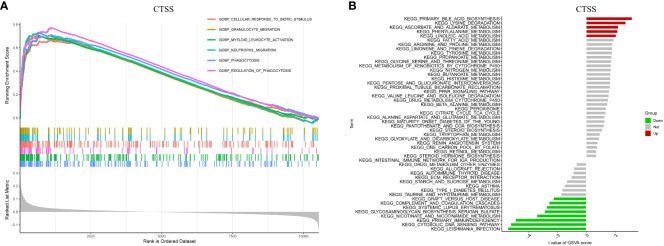
Biological processes and relevant mechanisms related to the CTSS expression. This figure explores the biological processes and relevant mechanisms related to the expression of CTSS. It includes GSEA analysis for biological processes **(A)** and GSVA analysis for relevant mechanisms **(B)** associated with CTSS.

To gain insights into the potential mechanisms underlying the role of CTSS in the progression of IgAN, we conducted GSEA and GSVA. The GSEA results highlighted significant enrichment of processes such as granulocyte migration, myeloid leukocyte activation, and neutrophil migration in IgAN patients exhibiting higher CTSS expression, as depicted in **Figure 8A**.

Furthermore, GSVA analysis unveiled a close association between elevated CTSS expression and the activation of diverse metabolic pathways, encompassing linoleic acid metabolism, lysine degradation, and phenylalanine metabolism. Conversely, it demonstrated a link between CTSS and the suppression of particular pathways, including the cytosolic DNA sensing pathway, nicotinate metabolism, and primary immunodeficiency, as visualized in **Figure 8B**. Both the findings from GSEA and GSVA imply that CTSS may play a role in the development of IgAN by influencing immune-related processes and metabolic pathways.

### 3.5 Analysis of immune cell infiltration

Given the pivotal role of immune dysregulation in the progression of IgAN and the association between elevated CTSS levels and an increased risk of IgAN development, we conducted an immune infiltration analysis utilizing the CIBERSORT algorithm. This analysis aimed to shed light on the relationship between CTSS and the immune cell infiltrates in renal tissues.

**Figure 9:**
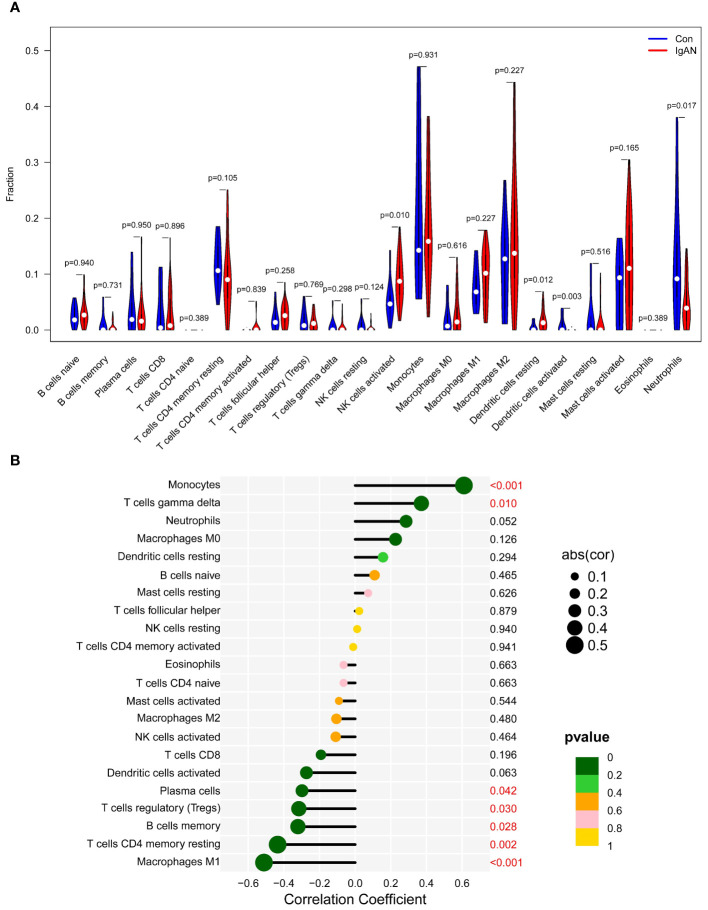
Analysis of immune cell infiltration. This figure provides an analysis of immune cell infiltration in IgAN patients and controls. It features a violin plot of immune cells with differential infiltration based on CIBERSORT **(A)** and correlations between CTSS expression and the extent of infiltration of immune cell subtypes **(B)**.

As illustrated in **Figure 9A**, we observed that activated NK and resting dendritic cells were more abundant in IgAN samples than in control samples. Conversely, activated dendritic cells and neutrophils were less prevalent. Importantly, the expression of exhibited a positive association with the infiltration of monocytes and T cells gamma delta, while concurrently showing negative correlations with plasma cells, Tregs, memory B cells, T cells CD4 memory resting, and macrophages M1 (**Figure 9B**; r=0.61, *P*<0.001; r=0.37, *P*=0.010; r=-0.30, *P*=0.042; r=-0.32, *P*=0.030; r=-0.32, *P*=0.028; r=-0.44, *P*=0.002; r=-0.51, *P*<0.001, respectively). The results of XCELL algorithm also showed that the expression of CTSS was positively correlated with the infiltration of 18 cell types, including neutrophils, in the IgAN renal tissues, as detailed in **Supplementary Figure 2**. These findings underscore the pivotal role of CTSS in the context of immune abnormalities observed in IgAN.

### 3.6 Molecular docking and virtual screening for potential therapeutic targets in IgAN

As previously shown, elevated levels of CTSS are associated with an increased risk of IgAN, and this enzyme is notably expressed in both serum and renal tissues of IgAN patients. These observations imply that CTSS may serve as a vital therapeutic target for IgAN. Despite this potential, no drugs targeting CTSS have been clinically approved to date. To bridge this gap, we engaged in molecular docking and virtual screening to identify potential therapeutic agents from the FDA-approved and pharmacopeia drug libraries, thereby leveraging their established safety profiles.

The FDA-approved and pharmacopeia drug library encompasses 3,158 compounds, and their detailed information is available in **Supplementary Table 3**. Our screening process involved several steps. Initially, we generated all stereoisomers for each ligand in a single conformation. After HTVS, we retained the top 50% of the best-scoring compounds for the subsequent step. In the second stage, we selected the stereoisomer with the best score for each ligand, generating only one conformation per stereoisomer. Following Glide SP docking, we retained the top 20% of the highest-scoring compounds for the third phase. In Glide XP, the top 100 compounds were chosen and subjected to Molecular Mechanics-Generalized Born Surface Area (MM-GBSA) analysis. Ultimately, 26 compounds with favorable Glide XP scores and MM-GBSA scores within the top 50 were identified as promising drugs. Their detailed information is available in **Supplementary Figure 3**.

**Figure 10:**
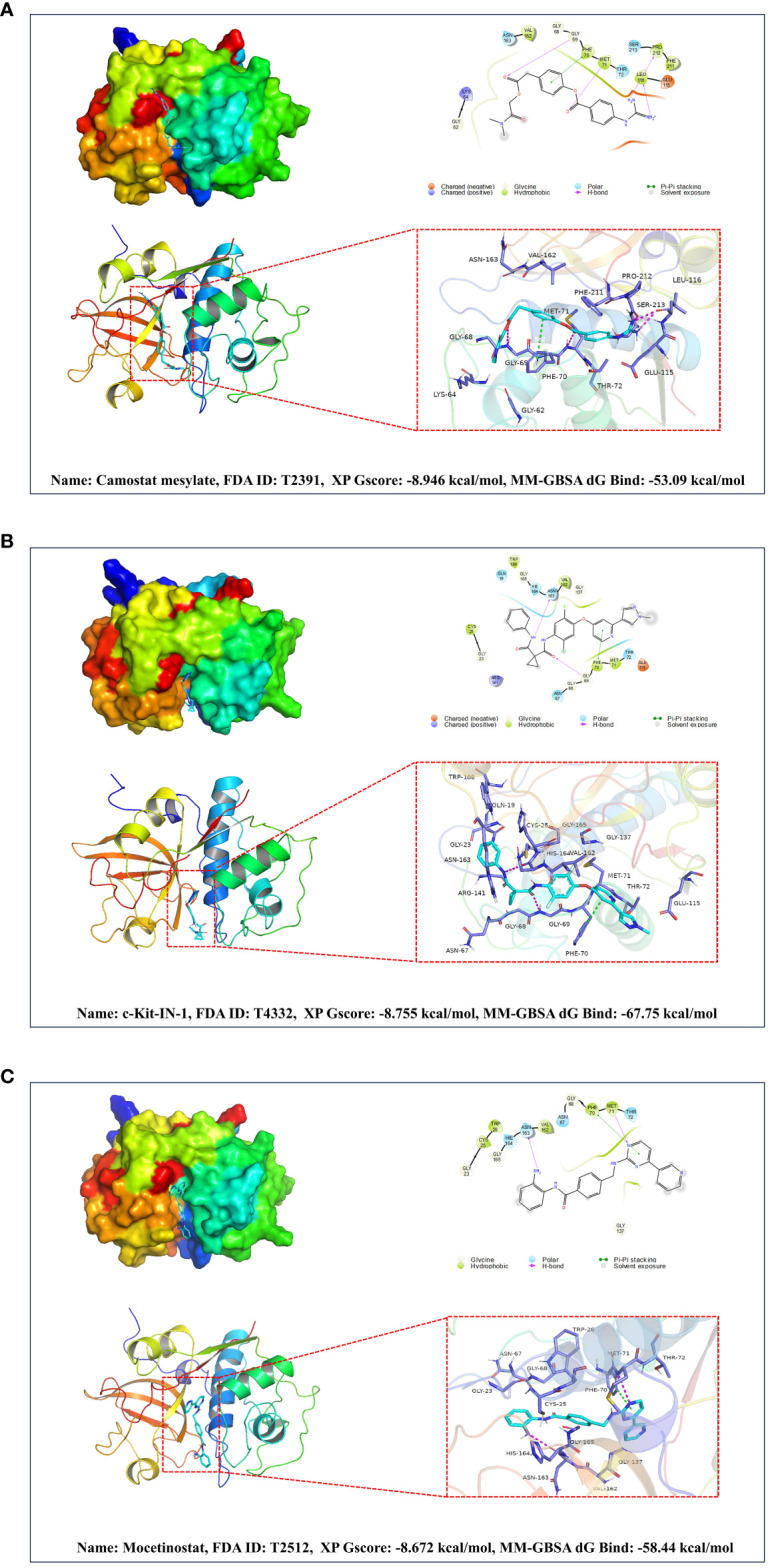
Based on molecular docking and virtual screening obtained the most promising targeted drugs for CTSS. This figure is centered around molecular docking and virtual screening to identify potential targeted drugs for CTSS. It includes molecular docking schematics of Camostat mesylate **(A)**, c-Kit-IN-1 **(B)**, and Mocetinostat **(C)** with CTSS. The figures depict 3D interactions, highlighting active ingredients, amino acids forming interactions, hydrogen bonds, and Pi-Pi stacking interactions.

Based on a combination of Glide XP scores, MM-GBSA scores, and clustering analysis, we identified Camostat mesylate, c-Kit-IN-1, and Mocetinostat as the three most promising drugs for targeting CTSS. Further details regarding their binding to CTSS are illustrated in **Figure 10**.

## 4 Discussion

Cathepsins, as a class of processing enzymes regulating protein trafficking and secretion, play an important role in the proteolytic events of many autoimmune diseases such as rheumatoid arthritis, systemic lupus erythematosus and Syogren’s syndrome (34–36). The development and progression of IgAN entail a complex process in which proteolytic events also play pivotal roles (37). Therefore, it is reasonable to hypothesize that cathepsins may exert important effects on the development and progression of IgAN as well. Extensive publicly available genetic data was explored in our study to systematically investigate the causal relationships between nine distinct cathepsins and the risk of IgAN. This study represents a groundbreaking MR analysis exploring the causal relationships between various cathepsins and IgAN. Through integrating results from both univariable and multivariable analyses, we have identified CTSS as a significant risk factor for IgAN, with no evidence of reverse causality for CTSS observed.

In recent years, there has been a growing interest in unraveling the pathophysiology of IgAN. The predominant hypothesis concerning IgAN pathogenesis posits a ‘four-hit’ process. This process involves the deposition of galactose-deficient IgA1-containing immune complexes (Gd-IgA1-ICs) in the glomerular mesangial areas, triggering the complement cascade, cellular proliferation, and the release of cytokines and chemokines, leading to subsequent glomerular injury (38). Additionally, aberrant activation of the complement system plays a critical role in the pathological progression of IgAN (39). However, the precise reasons why the kidney is primarily affected by this systemic abnormality remain not entirely understood. No specific IgAN biomarker has been validated for diagnosis, prognosis, or tracking the response to therapy (40).

The conclusions presented in this study establish the causal relationship between CTSS and IgAN. Cathepsin S is garnering increasing attention due to its distinctive characteristics compared to other cathepsin family members. It exhibits relatively limited expression and can maintain catalytic activity under neutral to mildly alkaline pH conditions. These properties have significant implications in regulating various health conditions, such as respiratory disease, pain, cardiovascular disease, diabetes/obesity, inflammation, autoimmune disease, cancer, and neuropathy (9, 10, 41–46).

The mechanism by which CTSS affects IgAN still requires deeper exploration. Studies have reported correlations between the expression of either HLA-DQB1 or HLA-DRB1 and the severity of IgAN (47–49). Cathepsin S plays a crucial role in processing the invariant chain/CD74 to facilitate MHC-II antigen presentation (50). Therefore, it is reasonable to speculate that CTSS influences IgAN by altering MHC antigen presentation. Moreover, CTSS has been shown to impact endothelial inflammation and complement protein activity under hyperglycemic conditions *in vitro* by inhibiting NF-κB signaling (51), suggesting a potential influence on IgAN via the complement system. Furthermore, it has been observed that CTSS plays a role in promoting the proliferation of glomerular mesangial cells, thereby contributing to the pathogenesis of IgAN (10). Recent research has also indicated that circulating CTSS levels correlate with GFR decline and levels of soluble tumor-necrosis-factor receptor (sTNFR) 1 and sTNFR2 in mice and humans, suggesting another potential pathway of influence (9). Nevertheless, the precise mechanisms through which CTSS impacts the progression of IgAN and modifies the ultimate phenotype remain subjects that necessitate further investigation and exploration.

Immune dysregulation stands as a significant mechanism in the development of IgAN, and accumulating evidence underscores the critical role played by infiltrating immune cells in kidney tissue in the pathogenesis of IgAN (52, 53).

Our study has revealed noteworthy correlations between CTSS expression and patterns of immune cell infiltration in IgAN renal tissues. We observed that CTSS expression positively correlates with the infiltration of monocytes and gamma delta T cells, while it negatively correlates with Tregs, memory B cells, macrophages M1, and CD4 memory resting T cells in IgAN renal tissues.

Research conducted by Cols et al. highlights the importance of diminished CD89 expression on nonclassical monocytes as an indicator of poorer renal prognosis in IgAN patients (54). The presence of gamma delta T cells has been associated with progressive IgAN (55, 56). Furthermore, plasma cells and B cells are implicated in exacerbating or contributing to IgAN by producing autoantibodies. Tregs, classified into Treg1 (CCR7 high, TCF7 high, and HLA-DR low) and Treg2 (CCR7 low, TCF7 low, and HLA-DR high) subsets, have shown associations with kidney injury recovery, with increased FOXP3+ Tregs potentially correlating with milder histological lesions (57). CD4+CD25+ Tregs have demonstrated the ability to improve symptoms in IgAN rat models (58). M1 macrophages have been associated with acute inflammatory responses in the early stages of IgAN, potentially leading to apoptosis and necrosis of normal histiocytes (59). Moreover, the presence of CD68+ macrophages, a marker of pan-macrophages or M1 type, has been positively correlated with serum creatinine, proteinuria, disease progression, and worse outcomes in IgAN patients (60). Notably, the results of our bioinformatics analyses support that increased CTSS is associated with higher neutrophils’s functional activation and higher infiltration in IgAN renal tissues. But in kidney biopsies, the diffuse high expression of CTSS in the brush border of renal tubules in IgAN was not observed to be accompanied by large numbers of neutrophil infiltrates. It may be due to the fact that the samples we collected were patients with clinically and pathologically definitive diagnoses of IgAN and would not reflect the very early stages of the disease, and it is possible that in the very early stage of IgAN onset, increased CTSS is accompanied with a transient abnormal infiltration and activation of neutrophils in the renal tissues, which is involved in the early pathological changes of IgAN, but it is just a conjecture. Altogether, CTSS may play a role in the progression of IgAN by influencing the infiltration of immune cells in renal tissue but the mechanism behind the involvement of CTSS in the development of IgAN is very complex, and more in-depth systematic studies are needed in the future.

Furthermore, we delved into the expression of CTSS in IgAN and observed a significant upregulation of this enzyme in both renal tissues and serum of IgAN patients compared to control subjects. This finding aligns with the research conducted by Zhao et al (10). Notably, Zhao et al.’s study focused on children, whereas our investigation marks the first examination of CTSS expression in adult IgAN patients.

The management of IgAN presents a substantial challenge. Apart from the use of mycophenolate mofetil specifically in patients of Asian descent, there is scarce supporting evidence regarding the effectiveness of supplementary immunosuppressive treatments beyond corticosteroids (61). Several clinical trials, encompassing interventions ranging from enteric-coated budesonide to B-cell function blockade and complement inhibitors, show potential for targeted treatment of high-risk IgAN patients. However, the precise effects of these interventions require further investigation (62). Consequently, it holds paramount importance to explore novel therapeutic options for IgAN.

Because CTSS has been identified as a potential contributor to IgAN through systematic MR analysis and exhibits significant overexpression in renal tissues and serum of IgAN patients, it presents an exciting prospect as a therapeutic target. This discovery suggests that drugs targeting CTSS may hold promise for IgAN treatment. It is well-established that conventional drug development is a resource-intensive, high-risk, and time-consuming process. In contrast, drug repositioning involves repurposing existing pharmaceutical ingredients that are already approved for one purpose for a new indication (63). Compared to traditional drug development, drug repositioning boasts advantages such as lower research and development costs, relatively known safety profiles, and shorter development timelines, garnering increased attention (64).

Considering the absence of approved targeted drugs against CTSS for clinical use, we conducted molecular docking and virtual screening to identify potential candidates from the FDA-approved and pharmacopeia drug library for drug repositioning. After rigorous screening, Camostat mesylate, c-Kit-IN-1, and Mocetinostat emerged as the three most promising drugs for targeting CTSS. However, their exact effects necessitate further validation.

In this study, we utilized data from extensive GWAS cohorts and applied genetic variants in bidirectional and multivariate MR analyses to thoroughly examine the causal effects of various cathepsins on IgAN. By incorporating both multivariate and reverse MR analyses, we minimized confounding factors, enhancing the robustness of our causal inferences. Moreover, our research confirmed the expression of key cathepsins in IgAN and explored the underlying mechanisms using bioinformatics. Additionally, we pursued the identification of potential therapeutic drugs through drug repositioning, thus paving new paths for the diagnosis and treatment of IgAN.

However, it is crucial to acknowledge certain limitations in our study. First, the participants included are exclusively of European descent, which may limit the applicability of our findings to other ethnic groups. Second, our investigation did not extend to exploring the relationship between CTSS and the prognosis of IgAN patients. Lastly, we did not perform experimental validations for these candidates while identifying potential drugs targeting CTSS.

## 5 Conclusion

Our investigation has revealed convincing evidence linking elevated levels of CTSS with an increased risk of IgAN. We noted a significant upregulation of CTSS in both the serum and renal tissues of IgAN patients. These findings could prove invaluable as markers for the diagnosis and prognosis of IgAN. Furthermore, our exploration into inhibitors targeting CTSS presents a promising pathway for developing effective treatments for IgAN.

## Data availability statement

The data presented in the study are deposited in the Figshare repository at the following link: https://doi.org/10.6084/m9.figshare.26056969.v2.

## Ethics statement

This study was approved by the ethical committees of the First Hospital of Jilin University, under approval number 2023-508. The studies were conducted in accordance with the local legislation and institutional requirements. The participants provided their written informed consent to participate in this study.

## Author contributions

SF: Investigation, Software, Writing – original draft. MW: Formal analysis, Funding acquisition, Writing – original draft. YC: Formal analysis, Funding acquisition, Methodology, Writing – original draft. YG: Writing – review & editing, Resources. JY: Writing – review & editing, Investigation, Methodology. XW: Validation, Writing – review & editing, Visualization. SS: Validation, Writing – review & editing, Supervision. HW: Writing – review & editing, Formal analysis, Investigation, Project administration, Validation. FM: Writing – review & editing, Software. YZ: Resources, Writing – review & editing. SW: Writing – review & editing, Methodology. HX: Funding acquisition, Project administration, Resources, Visualization, Writing – original draft. ZX: Formal analysis, Funding acquisition, Resources, Visualization, Writing – review & editing.
