# Supplementary material for: Cathepsin S (CTSS) in IgA nephropathy: an exploratory study on its role as a potential diagnostic biomarker and therapeutic target
Source: Front Immunol. 2024 Jun 24;15:1390821. doi: 10.3389/fimmu.2024.1390821 (PMC11229174; doi:10.3389/fimmu.2024.1390821)
Supplement: Supplementary file 1 [file DataSheet_1.docx]

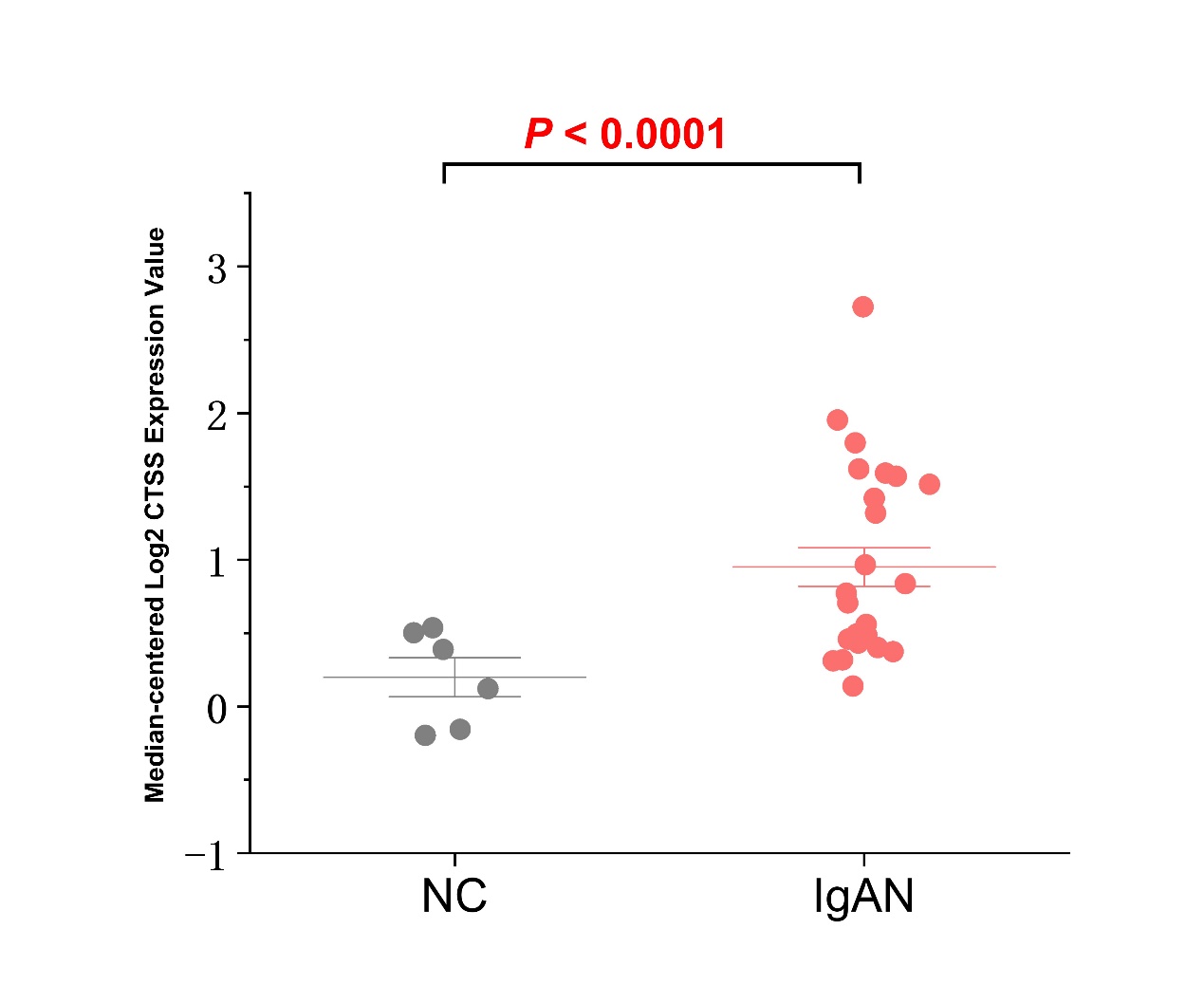


**Supplementary Figure 1:** Validation of CTSS expression in a small sample cohort from Canada including 27 IgAN patients and 6 healthy pre-transplant living donor controls with renal tissue microarray data.


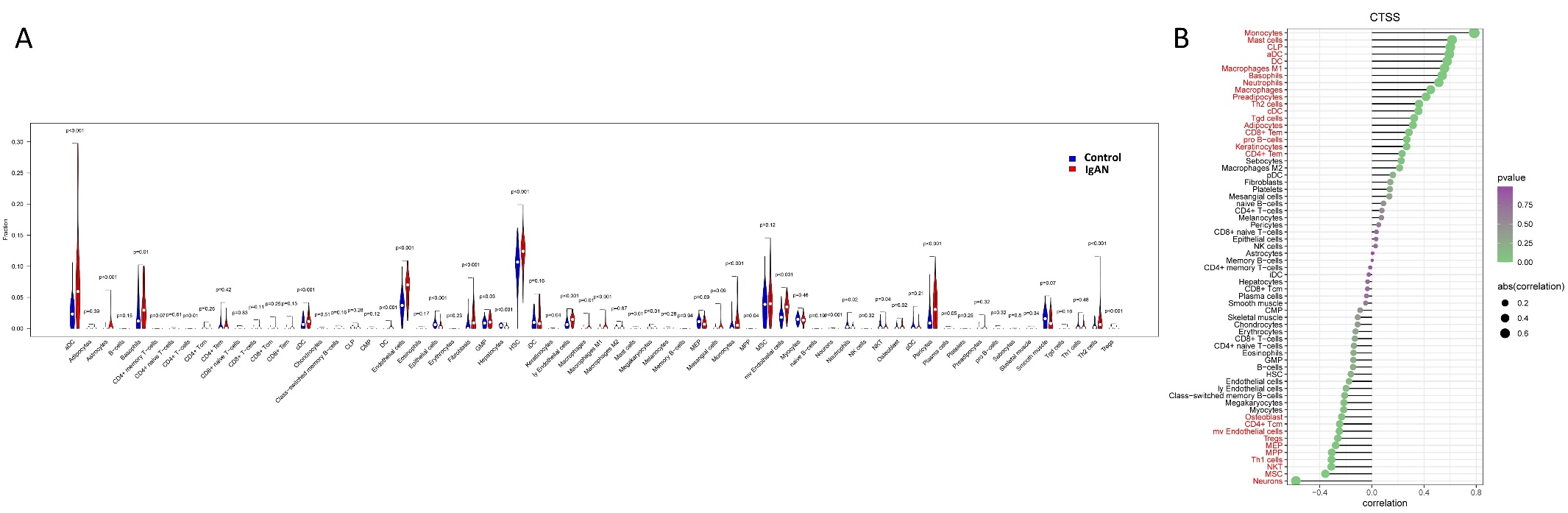


**Supplementary Figure 2: Analysis of immune cell infiltration.** This figure provides an analysis of immune cell infiltration in IgAN patients and controls. It features a violin plot of immune cells with differential infiltration based on XCELL (A) and correlations between CTSS expression and the extent of infiltration of immune cell subtypes (B).


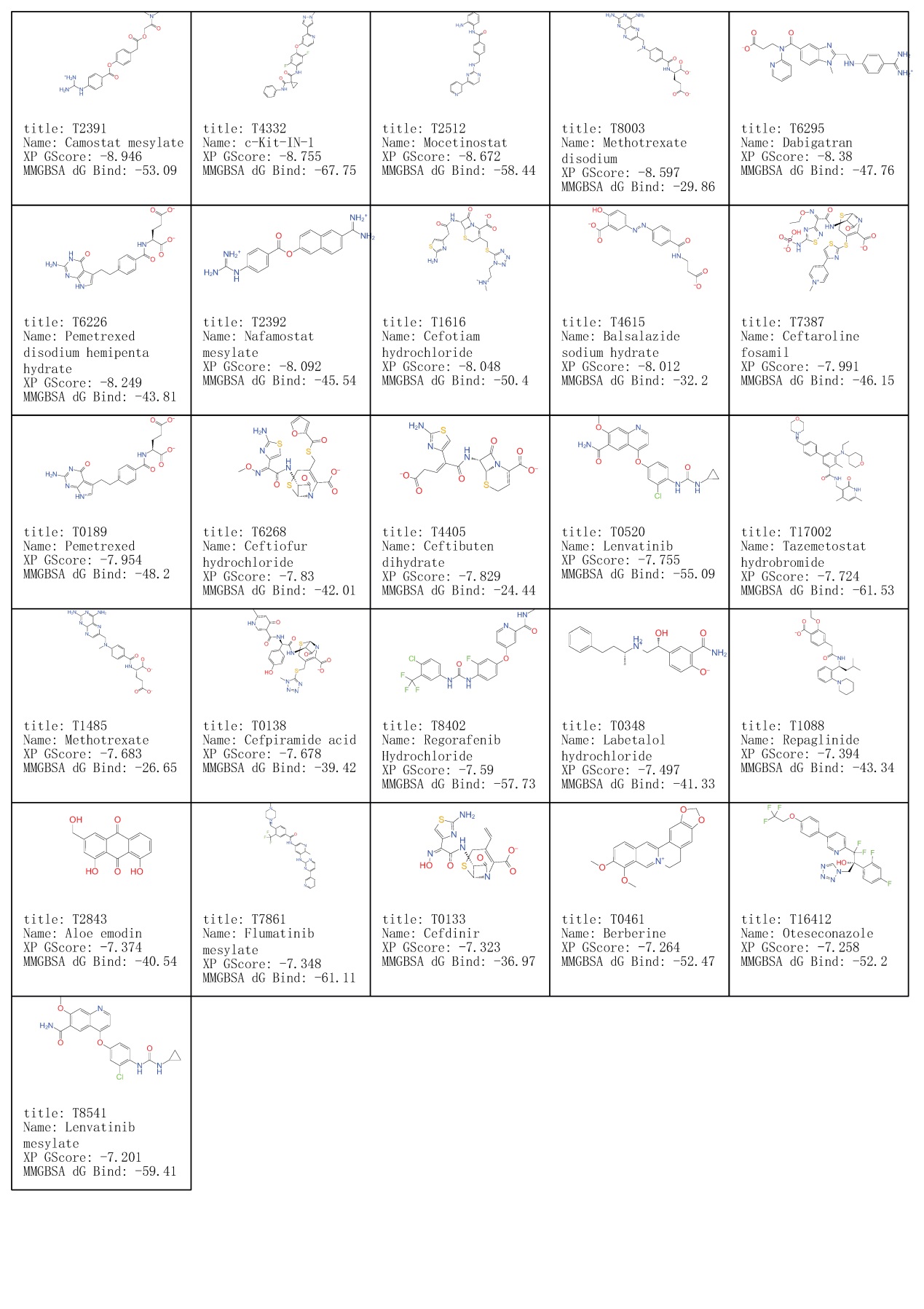


**Supplementary Figure 3:** Detailed information of 26 compounds with Glide XP scores and MM-GBSA scores in the top 50 screened after virtual screening.
